# Corrigendum

**DOI:** 10.1113/EP091585

**Published:** 2023-10-20

The order of affiliation “Student Research Committee, Kerman University of Medical Sciences, Kerman, Iran” was changed from 1st to 8th in position in the published article but the correct order is:

1. Student Research Committee, Kerman University of Medical Sciences, Kerman, Iran
2. Department of Physiology and Pharmacology, Kerman University of Medical Sciences, Kerman, Iran
3. Physiology Research Center, Institute of Pulmonary Physiology, Kerman University of Medical Sciences, Kerman, Iran
4. Faculty of Physical Education and Sports Sciences, Department of Exercise Physiology, Shahid Bahonar University of Kerman, Kerman, Iran
5. Non communicable Diseases Research Center, Bam University of Medical Sciences, Bam, Kerman, Iran
6. Faculty of Medicine, Department of Medical Immunology, Rafsanjan University of Medical Sciences, Rafsanjan, Iran
7. Pathology and Stem Cell Research Center, Department of Pathology, Afzalipour School of Medicine, Kerman, Iran
8. Legal Medicine Research Center, Legal Medicine Organization, Kerman, Iran

In the “Additional information” section of the article, under the heading “Funding information”, the funder was incorrectly listed as “Physiology Research Center, Institute of Neuropharmacology, Kerman University of Medical Sciences” and the Funding section should correctly read as:

## FUNDING INFORMATION

“Student Research Committee, Kerman University of Medical Sciences, Kerman, Iran”.

Furthermore, under the heading “Acknowledgements”, the funding number was not provided and the “Acknowledgements” section should read as follows:

The authors apologize for these errors.
